# Customized SNP Panel for Local Brazilian Sheep Breed Assignment

**DOI:** 10.3390/genes17090991

**Published:** 2026-08-24

**Authors:** Camila Souza Rodrigues, Danielle Assis de Faria, Samuel Rezende Paiva, Concepta McManus

**Affiliations:** 1 Faculty of Agronomy and Veterinary Medicine, Central Institute of Sciences, Darcy Ribeiro Campus, University of Brasília, Brasília 70910-900, DF, Brazil; rodriguesscamila@outlook.com (C.S.R.); concepta@unb.br (C.M.); 2 Embrapa Recursos Genéticos e Biotecnologia, Brasília 70770-917, DF, Brazil; samuel.paiva@embrapa.br

**Keywords:** genetic resources, population genomics, traceability, fixation index, random forest

## Abstract

**Background/Objectives:** Brazilian locally adapted sheep breeds represent valuable genetic resources. However, the commercial valorization of these breeds depends in part on genetic certification to support traceability and verify product origin. This study aimed to identify and evaluate a minimum set of highly informative SNPs for accurate and efficient breed assignment across five Brazilian locally adapted sheep breeds. **Methods:** A total of 677 samples were genotyped using the Embrapa Multispecies 65 K Illumina Infinium 1 chip, which contains 2926 markers for *Ovis aries*. The dataset was partitioned into training (*n* = 566) and independent testing (*n* = 111) sets. Markers were ranked according to genetic differentiation based on pairwise Wright’s fixation index (*F_ST_*) using the Toolbox for Ranking and Evaluation of SNPs (TRES), generating three nested reduced panels of 288, 192, and 96 SNPs. Panel performance and preservation of population structure were evaluated using Random Forest classification, Principal Component Analysis (PCA), and ADMIXTURE. **Results:** The 96-SNP panel achieved classification accuracy comparable to that of the 2145-SNP post-QC baseline and the 192- and 288-SNP panels (Cochran’s *Q* test: *Q* = 6.00, *df* = 3, *p* = 0.112), with no statistically significant difference in classification performance despite the substantial reduction in marker number. PCA and ADMIXTURE analyses indicated that the 96-SNP panel preserved the major population structure observed with the full post-QC marker set. **Conclusions:** Although formal analytical validation of a dedicated low-density genotyping assay remains necessary before routine implementation, the in-silico marker selection and empirical validation performed here provide an evidence-based framework for translating high-density genomic information into accessible and cost-effective applications for breed assignment, traceability, and conservation of Brazilian locally adapted sheep genetic resources.

## 1. Introduction

Sheep (*Ovis aries*) are among the earliest domesticated livestock species, possessing a rich evolutionary history shaped over millennia by artificial selection, human-mediated dispersal along ancient migration routes, and evolutionary adaptation to diverse environments [1,2]. The sheep populations currently present in Brazil reflect this complex historical continuum, originating from Iberian and African livestock introductions that subsequently underwent extensive diversification and local adaptation across the country’s distinct biomes [3]. For example, locally adapted hair-sheep breeds, such as the Brazilian Somali, Morada Nova, and Santa Inês, originated in the semi-arid Northeast, developing high heat tolerance and rusticity [3]. Conversely, wool-producing locally adapted populations, such as the Crioula in the southern pampas and the Pantaneiro genetic group in the central-western wetlands, reflect distinct evolutionary trajectories driven by environmental pressures unique to their respective ecosystems [4,5].

These locally adapted sheep constitute vital genetic resources, particularly for smallholders across the Northeast and South regions, contributing to sustainable meat, milk, leather, and wool production [6,7]. Crossbreeding with highly productive commercial breeds can offer immediate economic advantages by enhancing traits such as growth rate, carcass yield, and prolificacy. However, when conducted indiscriminately and without systematic breeding control, repeated crossbreeding leads to extensive genomic admixture and the erosion of breed-specific allele combinations [8,9,10]. This uncontrolled genetic dilution severely threatens conservation efforts, as it compromises the unique adaptive traits, such as heat tolerance, parasite resistance, and rusticity, which enable these breeds to thrive in marginal environments. Consequently, cost-effective molecular tools for accurate breed assignment are urgently needed to monitor population integrity, safeguard purebred conservation nuclei, and support genetic traceability programs.

Traditional traceability approaches based on pedigree records and phenotypic characterization are often unreliable in locally managed or smallholder production systems, where systematic recording is limited or absent [11,12]. In this context, genomic markers, such as single nucleotide polymorphisms (SNPs), have emerged as effective tools for accurate breed assignment in domesticated animal populations [13,14,15,16,17,18,19,20,21,22]. Although high-density SNP arrays are widely used for these purposes, their high cost and intensive computational requirements restrict routine application in large-scale monitoring, particularly in developing countries [23].

To address these limitations, reduced SNP panels composed of ancestry-informative markers (AIMs) have been developed as cost-effective alternatives for breed assignment across livestock species [24,25,26,27]. However, while global efforts have advanced sheep genomic traceability and characterization using minimal marker sets [21,28,29,30], most studies have focused on commercial or exotic breeds, leading to the underrepresentation of Neotropical sheep populations. This highlights a critical gap, as Brazilian sheep present complex demographic histories driven by admixture, drift, and selection [3], as well as documented breed-specific variation in loci controlling traits such as prolificacy and coat color, highlighting the distinct genetic architecture of Brazilian breeds relative to commercial European breeds [31]. Collectively, these findings underscore the need for a cost-effective and efficient SNP panel tailored to capture the breed-specific genomic structure of Brazilian locally adapted breeds.

In Brazil, the Embrapa Multispecies 65 K Illumina Infinium 1 chip was developed as a cost-efficient genotyping platform comprising 66,413 SNPs across 27 plant and animal species relevant to national agricultural resources, with its applicability previously demonstrated across crop and livestock studies [32,33,34]. For *O. aries*, the platform contains a defined subset of 2926 ovine-specific SNPs curated directly from commercial 50 K and high-density ovine arrays (Illumina and Neogen) to maximize genomic coverage across Brazilian sheep populations. While multispecies array architectures require careful consideration of probe specificity, Illumina Infinium 50-mer locus-specific hybridization chemistry inherently minimizes cross-species signal interference. Furthermore, to ensure data quality, all analyses in the present study were strictly restricted to the *O. aries* marker subset and subjected to stringent post-genotyping quality-control filtering. From this species-specific baseline, candidate markers were evaluated and ranked according to their discriminatory power for breed assignment, enabling the creation of progressively reduced AIM panels. Building on this platform, this study aimed to identify and empirically evaluate compact sets of highly informative SNPs capable of enabling accurate and efficient breed assignment in locally adapted Brazilian sheep, thereby establishing a validated framework for low-cost genomic tools to support breed traceability, origin certification, and genetic conservation.

## 2. Materials and Methods

### 2.1. Biological Samples

DNA was extracted from 677 whole-blood samples (preserved in EDTA) retrieved directly from the *Banco de DNA e Tecidos Animais* biobank at Embrapa Genetic Resources and Biotechnology. Because this study relied exclusively on archived biobank specimens and involved no live animal handling or primary tissue collection, formal ethical approval was not required. The sample set comprised individuals representing five principal locally adapted sheep breeds in Brazil, spanning both hair and wool coat types across diverse production systems (Table 1). To ensure reference purity for downstream AIM selection and validation, all candidate samples were derived from documented conservation nuclei and had been previously verified via genome-wide profiling. The dataset was then partitioned into training (*n* = 566) and independent testing (*n* = 111) sets.

Genomic DNA was extracted using the Gentra Puregene Blood Kit (Qiagen, Germantown, MD, USA; https://www.qiagen.com/us, accessed on 27 April 2026), following the manufacturer’s instructions. DNA quality and concentration were assessed by electrophoresis on a 1% agarose gel stained with ethidium bromide using lambda DNA standards (50, 100, and 200 ng/µL), and by spectrophotometric analysis (NanoDrop™ 8000, Thermo Fisher Scientific, Waltham, MA, USA; https://www.thermofisher.com/, accessed on 27 April 2026).

### 2.2. Genotyping and Quality Control (QC)

All samples used in this study were genotyped using the Embrapa Multispecies 65 K Illumina Infinium 1 chip (Neogen/GeneSeek, Lincoln, NE, USA, https://www.neogen.com/, accessed on 27 April 2026). This genomic tool was developed from the OvineSNP50 Genotyping BeadChip (Illumina Inc., San Diego, CA, USA, https://support.illumina.com/array/array_kits/ovinesnp50_dna_analysis_kit.html/, accessed on 27 April 2026). Embrapa Multispecies 65 K Illumina Infinium 1 comprises 2926 SNPs distributed across the 26 ovine autosomal chromosomes anchored in the sheep reference genome assembly ARS UI_Ramb_v3.0 (*O. aries*; NCBI RefSeq accession GCF_016772045.2). Genotype calling was performed using GenomeStudio 2.0 (Illumina Inc., San Diego, CA, USA, https://www.illumina.com/, accessed on 27 April 2026), following standard quality control procedures, and alleles were exported in the TOP format (A, C, G, T).

Quality control of the genotype data was performed using PLINK v1.9 [35]. Only autosomal SNPs were used, and the markers were filtered using the following criteria: call rate ≥ 95%, minor allele frequency (MAF) ≥ 0.05, Hardy–Weinberg equilibrium (HWE; *p* > 1 × 10^−6^), and linkage disequilibrium (LD) pruning. Pairwise LD was estimated using the r^2^ statistic implemented in PLINK, and pruning was performed using a sliding-window approach (50-SNP window, 5-SNP step size) with an r^2^ threshold of 0.20.

### 2.3. SNP Selection

After QC, individuals from each breed were allocated to independent training (*n* = 566) and testing (*n* = 111) datasets (Table 1). To identify ancestry-informative markers (AIMs) for breed assignment, candidate SNPs from the training dataset were evaluated using pairwise Wright’s fixation index ($F_{ST}$) implemented in the Java-based Toolbox for Ranking and Evaluation of SNPs (TRES) [36]. TRES computed *F_ST_* across all 10 pairwise comparisons among the five breeds, deriving a single ranking score per SNP equal to the unweighted arithmetic mean of its pairwise *F_ST_* values. Candidate SNPs were then ranked in descending order by this score, prioritizing markers with the highest overall genetic differentiation across breeds. The independent testing dataset was strictly reserved for panel validation. Based on this ranking, three nested panels comprising the top-ranked 96, 192, and 288 SNPs were intentionally selected to match standard low-density genotyping assay capacities (e.g., KASP and microfluidic/nanofluidic array platforms) for routine commercial application.

### 2.4. Breed Classification and Statistical Analysis

Population classification was performed using a Random Forest classifier implemented with the *ranger* [37] and *caret* [38] packages in R version 4.5.2 [39]. SNP genotype data from the training and independent testing datasets were aligned to ensure marker consistency, and marker nomenclature was standardized prior to model fitting. Missing genotypes were imputed using the modal genotype at each locus prior to model training.

Model hyperparameters were optimized exclusively on the training dataset through a repeated stratified 10-fold cross-validation procedure (10 folds, 3 repetitions), using a grid search. This optimization identified the optimal combination of *mtry*, *splitrule*, and *min.node.size* based on the highest mean balanced accuracy, while fixing the number of trees at 1000. The final Random Forest classifier was subsequently trained on the complete training dataset using the optimized hyperparameters, and SNP importance was estimated via permutation importance. Model performance was evaluated on the independent testing dataset using a confusion matrix to derive overall accuracy, Cohen’s Kappa coefficient, and balanced accuracy. To evaluate the impact of marker density reduction relative to the full, unselected SNP set, classification models were fitted independently for the three reduced panels (288, 192, and 96 SNPs) and the full 2145-SNP reference baseline. Posterior class probabilities were obtained for all test individuals; predictions with a maximum probability below 0.70 were flagged as “Unassigned” for exploratory analyses.

To independently evaluate whether the reduced SNP panels preserved the underlying population genetic structure, model-based ancestry estimation was performed using ADMIXTURE [40]. The independent testing dataset was analyzed separately for each of the three reduced SNP panels and the full post-QC 2145-SNP reference baseline under an unsupervised maximum-likelihood framework. The number of ancestral populations (*K*) was explored from 2 to 14, and the optimal *K* was determined using the cross-validation (CV) procedure implemented in ADMIXTURE, whereby the model exhibiting the lowest CV error was considered the best-supported solution. Individual ancestry coefficients (*Q-values*) were subsequently estimated for the optimal *K* and used to assess the ability of each reduced panel to recover the genetic structure and ancestry profiles of the reference breeds relative to the complete baseline. The individual ancestry coefficients were visualized using the R package *pophelper* v2.3.1 [41], which was also used to align cluster labels across the four SNP density panels (alignK function), ensuring that the same ancestral component was represented by the same color in all panels.

Additionally, Principal Component Analysis (PCA) was performed using PLINK v1.9 [35] to evaluate whether the reduced SNP panels preserved the major patterns of genetic structure and breed differentiation observed in the reference dataset. Separate PCAs were conducted for each reduced panel density (288, 192, and 96 SNPs) and the full 2145-SNP reference baseline using the independent testing dataset. The proportion of variance explained by each principal component was calculated from the eigenvalues generated by PLINK. Individual coordinates for the first two principal components (PC1 and PC2) were visualized using the R *ggplot2* package [42] to assess whether progressively smaller SNP subsets preserved breed-specific clustering patterns.

To compare classification accuracy among the full 2145-SNP reference baseline and the three reduced SNP panels (288, 192, and 96 SNPs), individual-level classification outcomes (correct/incorrect) from the Random Forest models were treated as paired binary data, given that the same animals were evaluated across all subsets. An omnibus Cochran’s *Q* test was performed to assess whether classification accuracy differed among all panels, followed by pairwise McNemar’s tests with continuity correction for *post hoc* comparisons. Resulting *p*-values were adjusted for multiple comparisons using the Holm method. All analyses were performed in R version 4.5.2 [39], using base R functions (*stats* package) and an implementation of Cochran’s *Q* test based on Cochran (1950) [43].

## 3. Results

### 3.1. Selection of Informative Markers and Genomic Distribution

**Figure 1:**
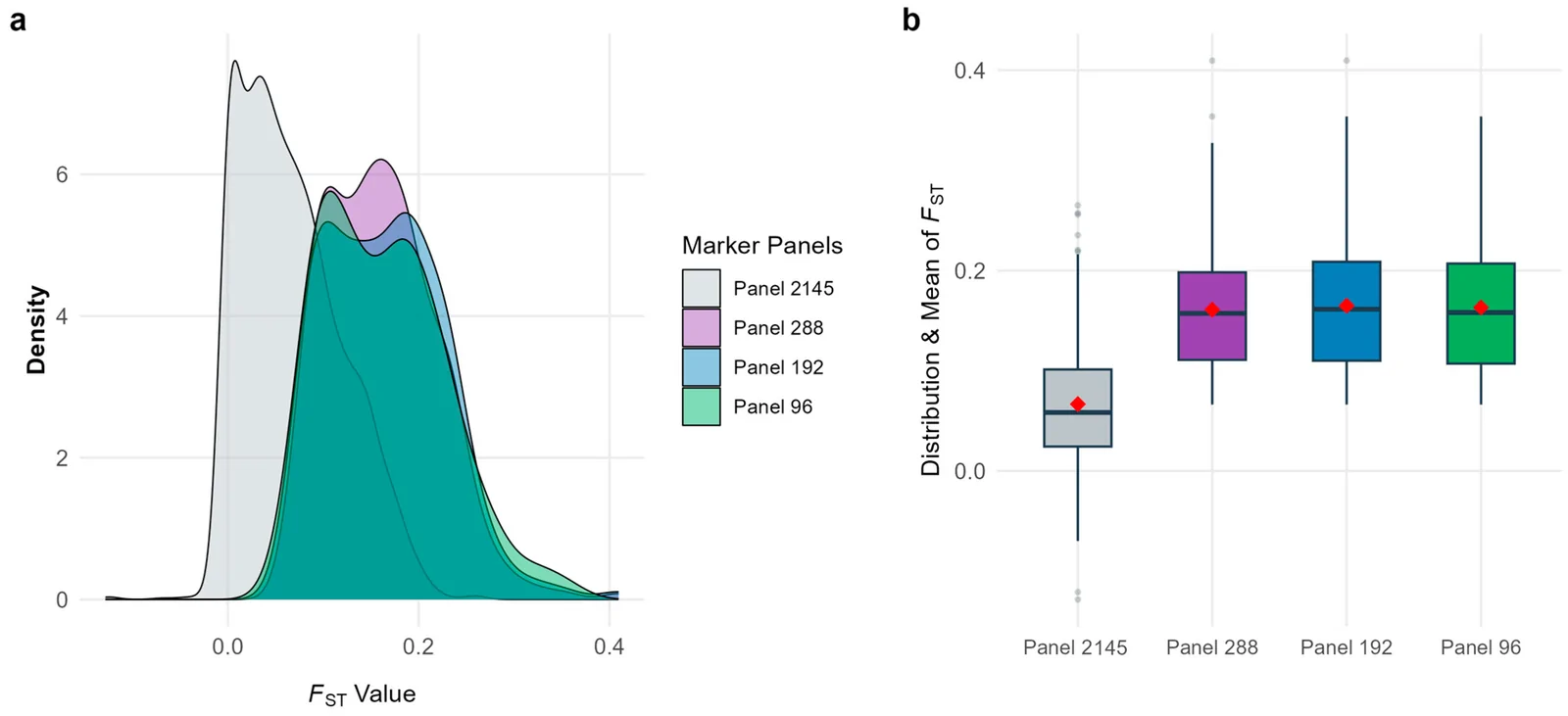
Distributional dynamics and enrichment of *F_ST_* values across different marker scales. (**a**) Probability density profiles showing the displacement toward higher differentiation of the three reduced panels compared to the full 2145-SNP reference baseline. (**b**) Boxplots of *F_ST_* values across the evaluated panels. Red diamonds indicate the mean *F_ST_* of each panel, whereas boxes, horizontal lines, and gray circles represent the interquartile range, median, and outliers, respectively.

Following quality control, 2145 out of 2926 initial SNPs were retained for downstream analyses. Subsequently, pairwise Wright’s fixation index (*F_ST_*) analysis identified the most informative loci for breed assignment, generating three nested reduced SNP panels containing the top 288, 192 and 96 markers. The *F_ST_* analysis demonstrated that the marker selection strategy effectively enriched the reduced panels with highly informative loci for breed discrimination (Figure 1).

Compared with the full post-QC 2145-SNP reference dataset, the reduced panels containing 288, 192 and 96 SNPs, respectively, presented an increase in *F_ST_* values, indicating a successful enrichment of loci with greater allele-frequency differentiation among breeds (Figure 1a). This pattern was further supported by the boxplot distributions (Figure 1b), in which all reduced panels showed substantially higher median and mean *F_ST_* values than the full 2145-SNP reference baseline, with a marginal increase in average *F_ST_* as panel size decreased. The three reduced subsets displayed highly consistent distribution shapes despite the progressive reduction in marker numbers, indicating that the sequential ranking procedure successfully retained the most informative markers.

**Figure 2:**
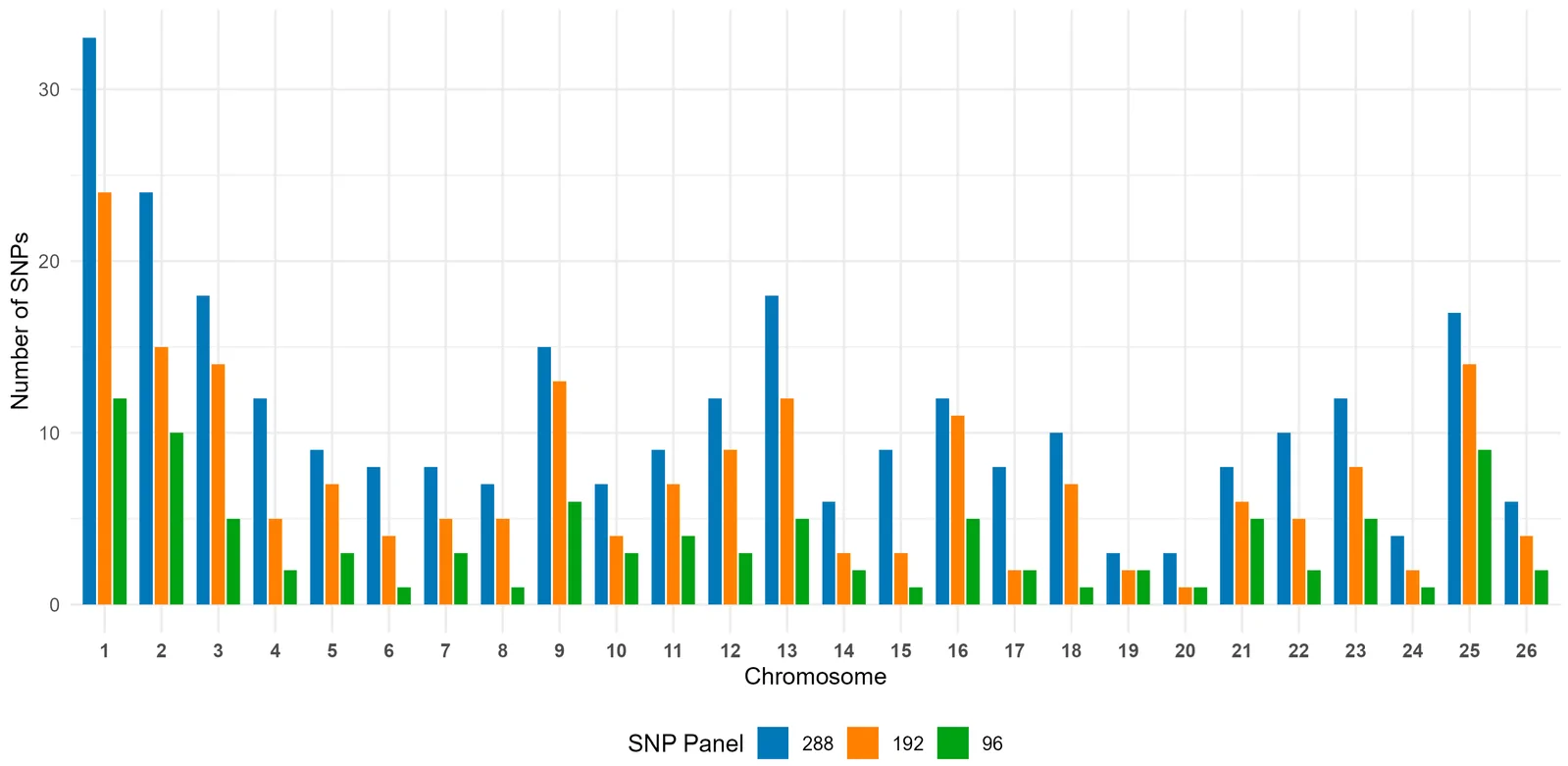
Chromosomal distribution of the selected SNPs across the three reduced panels.

The genomic distribution of the selected SNPs was subsequently evaluated to assess chromosome-wide representation. The selected markers were distributed across 26 sheep autosomes (Figure 2), maintaining broad genomic coverage despite progressive marker reduction. Notably, even the smallest panel (96 SNPs) retained at least one marker on every represented chromosome, indicating that marker reduction preserved genome-wide representation while substantially decreasing overall panel size. Detailed information on the SNPs included in each reduced panel, including marker identification, chromosomal location, and genomic position, is provided in Supplementary Table S1.

### 3.2. Principal Component Analysis (PCA)

**Figure 3:**
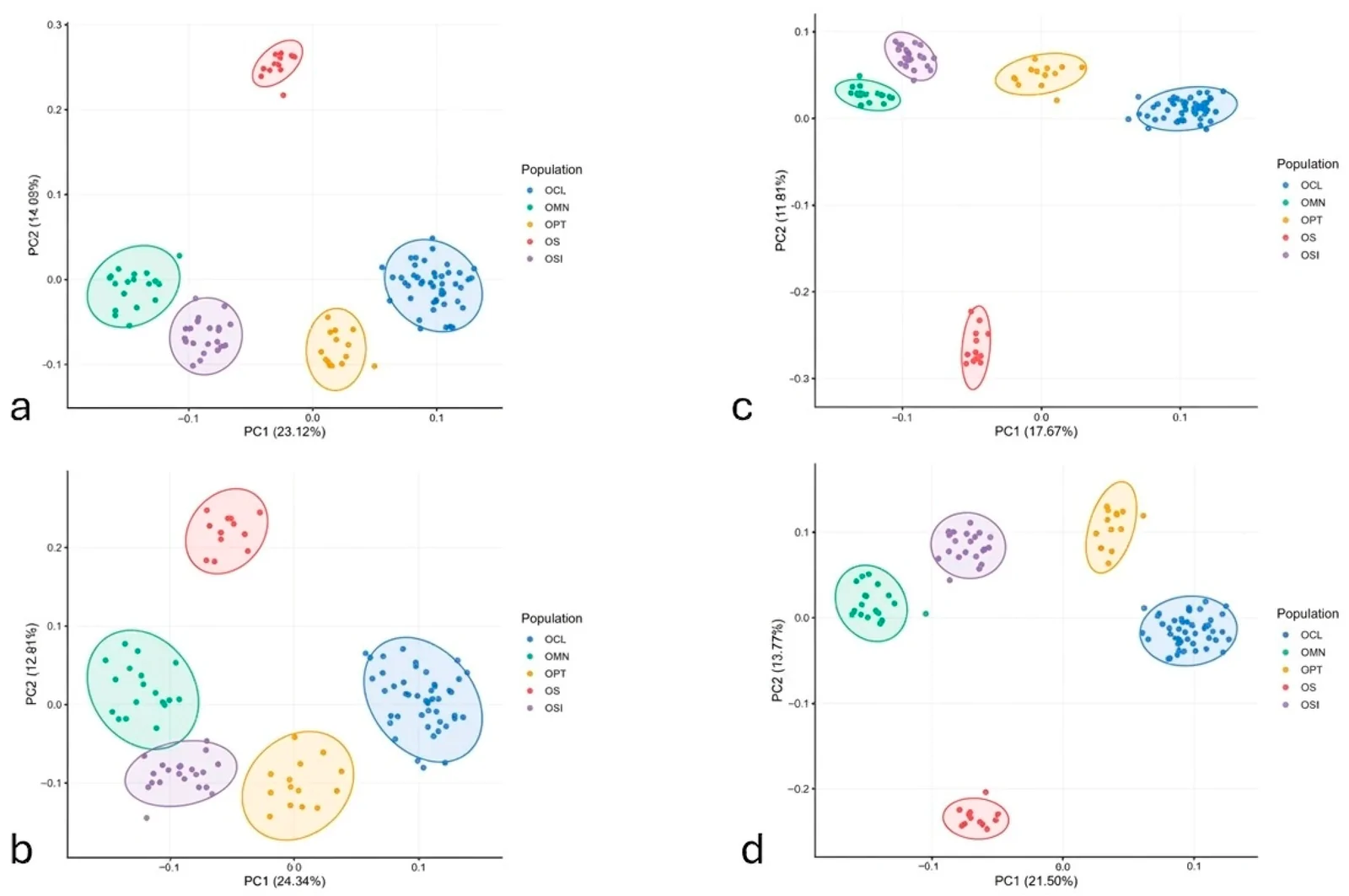
Principal component analysis (PCA) of the reduced SNP panels: (**a**) 96 SNPs, (**b**) 192 SNPs, and (**c**) 288 SNPs, compared with (**d**) the full post-QC 2145-SNP reference dataset. Breed codes are provided in Table 1.

Principal Component Analysis (PCA) was performed to evaluate whether the reduced SNP panels retained the genetic structure of the studied breeds following marker reduction. Overall, all three reduced panels preserved the major patterns of population differentiation observed in the full dataset. The first two principal components (PC1 and PC2) explained 23.12% and 14.05% of the total genetic variance for the 96-SNP panel (Figure 3a), 24.34% and 12.81% for the 192-SNP panel (Figure 3b), 17.67% and 11.81% for the 288-SNP panel (Figure 3c), compared with 21.50% and 13.77%, respectively, for the full 2145-SNP reference panel (Figure 3d). Despite the progressive reduction in marker count, the overall PCA topology remained highly consistent between the full dataset and the subset panels. This demonstrates that the targeted selection strategy successfully captured the key patterns of genetic differentiation among the evaluated breeds without the need for the full marker array.

### 3.3. Population Structure Inferred by Admixture

**Figure 4:**
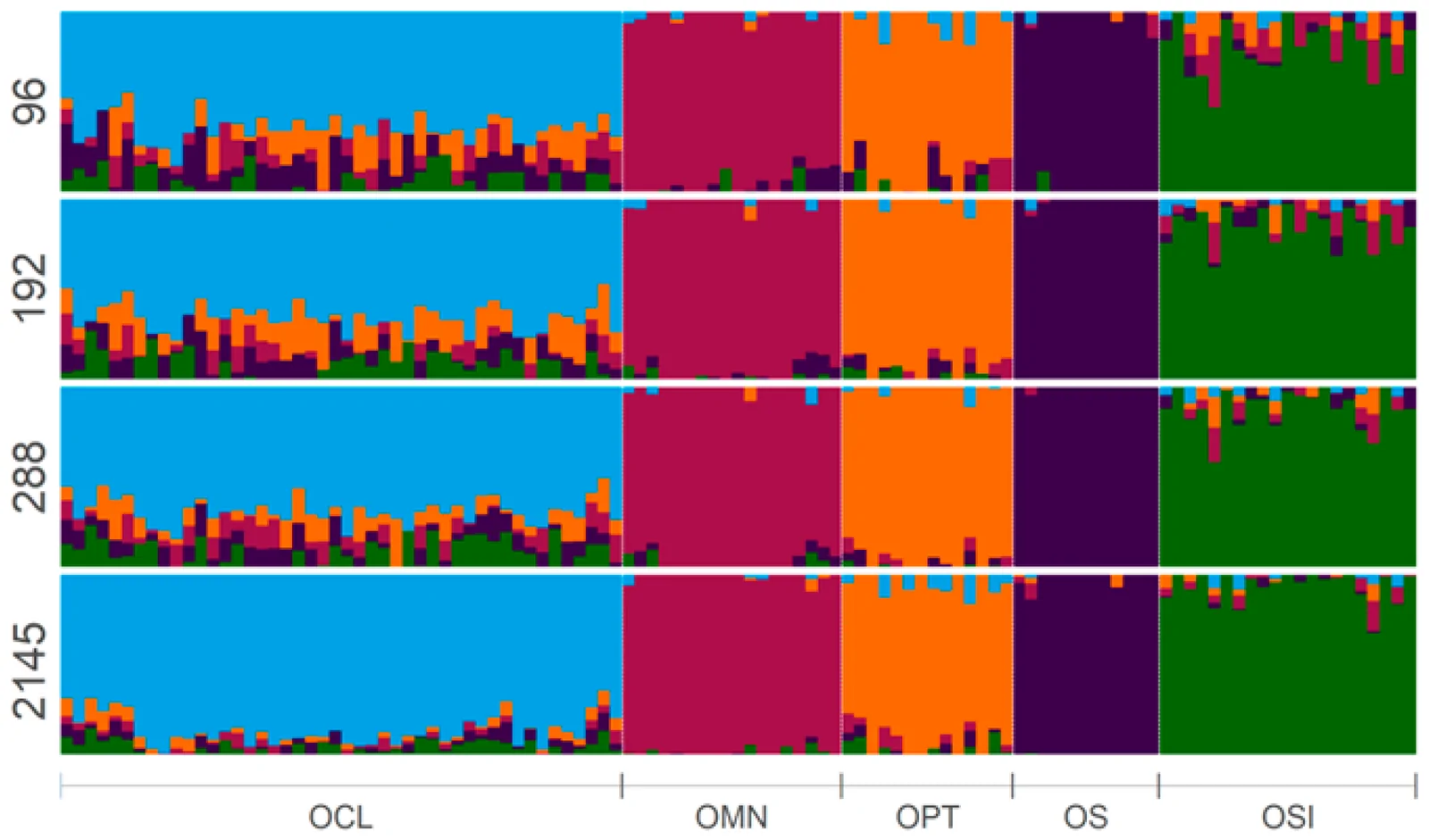
Population structure inferred by ADMIXTURE at the optimal number of ancestral populations (*K* = 5) for the three reduced SNP panels (96, 192, and 288 SNPs) compared to the complete 2145-SNP reference dataset. Individual ancestry proportions are represented by vertical bars, with colors indicating inferred ancestral clusters. Breed codes are listed in Table 1.

ADMIXTURE analyses consistently recovered the genetic structure of the five Brazilian sheep breeds across all three reduced, selected SNP panels when compared to the complete 2145-SNP reference baseline at *K* = 5 (Figure 4). The structure is supported by the lowest cross-validation (CV) error observed at *K* = 5 across all subsets and the full dataset (Supplementary Figure S1).

The 288-SNP panel produced highly homogeneous ancestry profiles with minimal evidence of admixture among breeds, closely recapitulating the baseline ancestry patterns. Similar clustering patterns were observed for both the 192-SNP and 96-SNP panels; although the 96-SNP panel showed a slight increase in background ancestry sharing between certain breeds, it still preserved well-defined, breed-specific clusters equivalent to the full reference baseline.

### 3.4. Random Forest Classification

The Random Forest classifier demonstrated consistently high predictive performance across all three reduced SNP panels (Table 2). The two intermediate-density panels (192 and 288 SNPs) exhibited the highest overall classification accuracy, correctly assigning 100% of the individuals to their respective breeds within the independent testing dataset (overall accuracy = 100%, balanced accuracy = 100%, Kappa = 1.000). In contrast, the 96-SNP panel showed only a marginal reduction in performance, maintaining an overall accuracy of 99.10%, a balanced accuracy of 99.17%, and an almost perfect agreement with reference classifications (Kappa = 0.988).

Conversely, the complete 2145-SNP baseline dataset presented the lowest predictive performance, yielding an overall accuracy of 97.30%, a balanced accuracy of 97.40%, and a Kappa coefficient of 0.963. The final model configurations obtained after hyperparameter optimization are provided in Supplementary Table S2.

Breed-specific performance metrics further confirmed the high discriminatory capacity of the targeted, reduced SNP subsets over the complete marker set (Table 3). Both the 192 and 288 SNP panels achieved flawless classification across all evaluated breeds, yielding sensitivity, specificity, positive predictive value (PPV), and negative predictive value (NPV) equal to 1.000 for every breed group. The minimal 96 SNP panel also maintained exceptional individual metrics, with mean sensitivity, specificity, PPV, and NPV of 0.986, 0.998, 0.991, and 0.998, respectively. Minor performance reductions in the 96-SNP panel were attributable to a single misclassification involving one Pantaneiro individual assigned to Santa Inês, resulting in a sensitivity of 0.929 for Pantaneiro and a PPV of 0.955 for Santa Inês.

The complete 2145-SNP dataset once again demonstrated lower comparative performance across individual populations, particularly for the Pantaneiro sheep, where sensitivity declined to 0.786, illustrating that in the present dataset, inclusion of the full post-QC marker set did not improve assignment performance relative to the targeted panels.

### 3.5. Comparative Evaluation of SNP Panels

Random Forest classification accuracy did not differ significantly among the four evaluated panels (2145, 288, 192, and 96 SNPs), as assessed by Cochran’s *Q* test (*Q* = 6.00, *df* = 3, *p* = 0.112). Pairwise *post hoc* comparisons using McNemar’s test with Holm adjustment revealed no statistically significant differences between any pair of panels. However, comparisons involving the full post-QC 2145-SNP reference dataset yielded the lowest raw *p*-values (2145- vs. 288-SNP and vs. 192-SNP panels: χ^2^ = 1.33, *p* = 0.248), aligning with the lower empirical test accuracy observed for the reference array (Table 2).

Furthermore, the difference in misclassification patterns between the 288-SNP and 96-SNP panels, or between the 192-SNP and 96-SNP panels, was not statistically significant after continuity correction (χ^2^ = 0, *p* = 1.000), despite a single discordant case. The 288-SNP and 192-SNP panels exhibited perfect classification agreement, which precluded McNemar’s test computation due to the total absence of discordant pairs. These results indicate that reducing marker density to 96 SNPs did not result in a statistically significant loss of breed assignment accuracy in the present test set, establishing this reduced panel as the optimal balance between cost-effectiveness and predictive performance.

## 4. Discussion

Brazilian locally adapted sheep breeds remain underrepresented in genomic traceability research, despite their vulnerability to extensive crossbreeding and the associated threat of genetic erosion. This study addresses this gap by evaluating progressively reduced SNP panels for breed assignment, demonstrating that high classification accuracy can be maintained using a compact set of highly informative markers. Three nested panels (288, 192, and 96 SNPs) were derived from an initial BeadChip dataset of 2145 post-QC SNPs. A comparative analysis revealed that all three reduced panels and the full post-QC 2145-SNP reference dataset achieved high classification accuracy, with no statistically significant differences among them (Cochran’s *Q* test: *Q* = 6.00, df = 3, *p* = 0.112). Given the comparable classification performance, the 96-SNP panel represents the most marker-efficient configuration, substantially reducing the number of markers required for reliable breed assignment.

Beyond reducing genotyping costs, lower marker density can also reduce computational requirements and analytical processing time, both of which are relevant to routine, large-scale applications. Because all selected markers are included in the commercially available Embrapa Multispecies 65 K Illumina Infinium 1 chip, the 96-SNP subset can also be identified retrospectively in existing datasets generated with this platform, without requiring additional laboratory procedures. In Brazil, genotyping costs vary according to batch size, laboratory, and processing conditions. Nevertheless, because the Multispecies platform consolidates multiple species on a single plate, it typically offers a lower per-sample cost than standard single-species commercial arrays. Although the selected markers are also present on standard ovine arrays, such as the OvineSNP50, the Multispecies platform offers additional operational flexibility by supporting genotyping across multiple livestock species within the same platform and reducing the dependence on large single-species sample batches. This flexibility may be particularly advantageous for small-scale producers and conservation programs managing relatively small or mixed-livestock populations.

Importantly, the proposed 96-SNP set is not presented here as a turnkey standalone assay, but rather as an empirically validated panel of Ancestry Informative Markers (AIMs) suitable for immediate in silico extraction. Furthermore, making these marker sets openly available provides a foundation for diagnostic laboratories or commercial companies to design ultra-low-cost targeted assays (e.g., KASP or microfluidic platforms) tailored for field deployment in routine breed assignment, population screening, and conservation programs.

In Brazil, the practical utility of this panel could directly support public institutions and breeding networks involved in livestock conservation. Embrapa (Brazilian Agricultural Research Corporation) maintains a nationwide network of conservation nuclei dedicated to safeguarding locally adapted livestock breeds, including sheep populations evaluated in this study. Accurate and affordable breed assignment can assist these programs in verifying the breed identity of animals maintained in conservation nuclei and monitoring unintended crossbreeding, an important concern for maintaining the genetic integrity of local genetic resources. Other organizations may also benefit from the availability of such a tool, including ARCO (*Associação Brasileira de Criadores de Ovinos*), as well as breed-specific associations representing locally adapted sheep, such as Morada Nova, Pantaneiro, and Crioula breeders, which may benefit from accessible approaches for breed verification and product traceability. By enabling accurate breed assignment with substantially lower genotyping and computational requirements than genome-wide approaches, the 96-SNP panel provides a practical and scalable basis for integration into conservation programs, regional breeding associations, and genetic resource management initiatives.

The high classification accuracy achieved by the reduced SNP panels reinforces the notion that successful breed assignment depends primarily on the discriminatory power of the selected markers rather than on absolute SNP density, as previously highlighted by [44]. The optimization strategy adopted here prioritized loci with high *F_ST_* values, thereby enriching the panels with ancestry-informative markers (AIMs) that capture substantial allele-frequency differentiation among breeds. Consequently, the classification performance of the optimized panels was comparable to that obtained with the full baseline array, despite drastically reducing marker density. This aligns with previous studies demonstrating that targeted, reduced SNP sets can effectively preserve population assignment accuracy [17,44,45].

Although the 288-SNP panel provided slightly clearer population separation in PCA space, this did not translate into superior machine learning assignment performance. The 192- and 96-SNP panels nevertheless preserved the major genetic structure observed in the PCA and ADMIXTURE analyses while achieving classification performance comparable to that of the larger panels. These findings suggest that increasing marker density beyond 96 SNPs provided limited additional benefit for breed assignment under the conditions evaluated here. Therefore, a relatively small set of highly informative markers can capture sufficient discriminatory signal to achieve reliable breed assignment, while substantially reducing the number of markers required [44,46].

Compared with previously published SNP panels for breed assignment and traceability in sheep, the optimized 96-SNP panel selected in this study achieved equivalent classification performance while requiring substantially fewer markers than many established arrays. For example, ref. [28] curated a 163-SNP panel validated primarily across cosmopolitan, commercial sheep breeds globally dispersed. Similarly, several other studies have reported high breed assignment accuracy using considerably larger SNP sets, typically ranging from 100 to over 6000 markers [21,30,47,48]. Despite the prevalence of larger arrays, minimal SNP panels have occasionally been implemented with success: [17] identified three breed-specific panels comprising 89 to 95 SNPs for Indian sheep breeds, while [45] achieved 100% classification accuracy using only 48 SNPs in local Sicilian breeds. However, direct cross-study comparisons must be interpreted with caution, as panel efficiency is inherently dictated by the underlying genetic differentiation among target populations, breed diversity, marker selection criteria, and classification algorithms. Collectively, our findings demonstrate that panel informativeness outweighs sheer marker volume, demonstrating that 96 carefully selected SNPs can accurately discriminate the Brazilian locally adapted sheep breeds evaluated here.

Crucially, while reduced SNP panels have been optimized for sheep breed assignment previously, these efforts have predominantly focused on European autochthonous or commercial breeds, leaving Neotropical populations markedly underrepresented in livestock genomic literature. Although Brazilian locally adapted sheep constitute invaluable genetic resources with unique physiological adaptations to tropical and semi-arid environments [3], no validated low-density SNP panel has previously been available for breed certification or traceability in these populations. Previous efforts, such as the 18-SNP panel proposed by [49] for Crioula, Morada Nova, and Santa Inês sheep, lacked experimental validation and were subsequently found to have insufficient discriminatory power when validated [50]. The present study addresses this long-standing gap by presenting an empirically validated, reduced 96-SNP panel, representing a valuable alternative with potential applications in breed certification, traceability, and conservation programs, particularly for Brazilian locally adapted sheep breeds suited to tropical and semi-arid environments, for which comparable genomic resources remain limited. Remarkably, this panel retained the major patterns of population structure even between closely related wool-producing populations (Crioula and Pantaneiro) with shared historical gene flow [5,6,9], as well as among hair-sheep breeds sharing African ancestral contributions [3].

The choice of 96, 192, and 288 SNPs was intentionally aligned with standard low-density genotyping formats, particularly the 96-well plate configuration commonly used for assays such as KASP and nanofluidic microarrays. Although smaller panels (e.g., 48 SNPs) are technically feasible, 96 markers were established as the minimum candidate threshold to maintain reliable breed-assignment performance. Previous ovine genomic studies have indicated that approximately 100 highly informative SNPs may be required to achieve robust individual assignment and adequately discriminate closely related livestock populations [47], while other studies have employed larger panels for breed assignment across a range of indigenous sheep breeds [21,28,30]. Thus, establishing 96 SNPs as the lower boundary provides a practical balance between genotyping cost and discriminatory performance.

From a practical perspective, the panel also has potential as a diagnostic tool for flagging admixed or crossbred individuals. Because the baseline reference populations were strictly defined using genetically verified purebreds, crossbred animals may be expected to show intermediate assignment probabilities or ambiguous classifications rather than high-confidence assignment to a single target breed. In commercial nucleus herds and breed associations, such patterns could help managers identify potential unintended crossbreeding, prevent admixed animals from entering purebred registries, and support the authenticity of origin-certified products. While precise quantitative estimation of ancestral proportions (i.e., fractional admixture breakdown) was beyond the scope of this study and warrants future validation in structured crossbred cohorts, the 96-SNP panel has the potential to fulfill an important operational role in distinguishing true purebred candidates from potentially admixed individuals.

This diagnostic precision is particularly impactful for smallholder production systems, which harbor the majority of Brazil’s locally adapted sheep but face substantial financial barriers to high-density genomic technologies [51,52]. Accurate and affordable breed assignment can directly support genetic certification and product traceability, potentially adding commercial value to artisanal meat, high-quality leather, specialized wool, and regional dairy products [7,53,54,55]. For instance, reliable breed-origin verification could contribute to geographical indication (GI) and other origin-based certification schemes for regional sheep products, as well as support premium differentiation of products from locally adapted breeds with documented adaptive traits. This potential is particularly relevant for breeds such as Pantaneiro, which are maintained under challenging environmental conditions and in multipurpose production systems [56,57,58]. Such applications may provide smallholder farmers with an additional means of differentiating their products based on verifiable genetic information.

Beyond commercial value-chain integration, the reduced SNP panel developed here offers a practical tool for conservation genetics by supporting breed identification, the identification of potentially admixed individuals, and the monitoring of population diversity [19,44,59]. This application may be particularly valuable for institutions such as Embrapa, which play a central role in the conservation and management of these genetic resources. In conclusion, the empirically validated 96-SNP panel provides an accessible and cost-effective framework to support the sustainable management, commercialization, and long-term conservation of Brazil’s locally adapted sheep genetic resources.

## 5. Conclusions

This study demonstrates that a targeted panel of 96 highly informative SNPs can accurately discriminate the Brazilian locally adapted sheep breeds evaluated here. Across the marker densities tested, the 96-SNP panel maintained classification performance comparable to that of larger SNP subsets and the full post-QC 2145-SNP reference dataset, highlighting the importance of marker informativeness over marker number for achieving reliable breed assignment.

Although formal analytical validation of a dedicated low-density genotyping assay remains necessary before field deployment, the in-silico marker selection and empirical validation performed here provide an evidence-based framework for translating high-density genomic information into accessible and cost-effective applications.

The resulting 96-SNP panel therefore represents a practical and scalable candidate tool for breed certification, genetic traceability, and breed-purity screening. By reducing the number of markers required for reliable assignment, the proposed panel has the potential to lower genotyping costs and facilitate the application of genomic tools in smallholder production systems and conservation programs. Its implementation could support institutional initiatives, including Embrapa’s conservation nuclei network, contributing to the sustainable management and long-term conservation of Brazil’s locally adapted sheep genetic resources.

## Figures and Tables

**Table 1:** Summary of the five locally adapted Brazilian sheep breeds evaluated in this study, including dataset partitioning for panel training and testing.

Breed	Breed Code	Sample Size Training/Testing	Coat Type *	Primary Purpose ^§^
Crioula	OCL	190/46	Wool	Triple-purpose (Meat, Wool and Skin)
Morada Nova	OMN	69/18	Hair	Dual-purpose (Meat/Leather)
Pantaneiro	OPT	55/14	Wool	Triple-purpose (Meat, Milk and Wool)
Brazilian Somali	OS	58/12	Hair	Dual-purpose (Meat/Leather)
Santa Inês	OSI	194/21	Hair	Dual-purpose (Meat/Leather)

* Coat Type: *Hair* denotes tropical, non-wool hair-sheep breeds characterized by natural shedding and high thermal tolerance; *Wool* denotes fleece-producing breeds that require shearing. ^§^ Primary Purpose: Refers to the main commercial or subsistence production purpose (e.g., meat, leather, wool, or dual-purpose).

**Table 2:** Random Forest classification performance for breed assignment across three reduced SNP panels versus the full post-QC 2145-SNP reference dataset, based on repeated 10-fold cross-validation and an independent test set (*n* = 111).

SNP Panel	Accuracy (95% CI) ^1^	Balanced Accuracy (%)	Kappa Coefficient	10-Fold CV Balanced Accuracy (%) ^2^	OOB Brier Score ^3^
96 SNPs	99.1 (95.08–99.98)	99.17	0.988	95.39	0.085
192 SNPs	100 (96.73–100)	100	1.000	95.52	0.092
288 SNPs	100 (96.73–100)	100	1.000	95.87	0.091
2145 SNPs	97.3 (92.30–99.44)	97.4	0.963	96.13	0.096

^1^ Values within parentheses indicate the lower and upper bounds of the 95% confidence interval for overall classification accuracy. ^2^ Mean balanced accuracy across repeated stratified 10-fold cross-validation (3 repeats), used as the model selection criterion. ^3^ Out-of-bag Brier score from the final Random Forest model, computed on the training set as a measure of probability calibration. All models were trained on the same 566 individuals and evaluated on the same independent test set of 111 individuals, using 1000 trees per Random Forest.

**Table 3:** Population-specific classification performance of the Random Forest model evaluated on an independent testing set (*n* = 111) comparing the three reduced SNP panels against the full post-QC 2145 SNP reference dataset.

SNP Panel	Breed	Sensitivity	Specificity	PPV ^1^	NPV ^2^
96 SNPs	OCL	1.000	1.000	1.000	1.000
	OMN	1.000	1.000	1.000	1.000
	OPT	0.929	1.000	1.000	0.990
	OS	1.000	1.000	1.000	1.000
	OSI	1.000	0.989	0.955	1.000
	Mean	0.986	0.998	0.991	0.998
192 SNPs	OCL	1.000	1.000	1.000	1.000
	OMN	1.000	1.000	1.000	1.000
	OPT	1.000	1.000	1.000	1.000
	OS	1.000	1.000	1.000	1.000
	OSI	1.000	1.000	1.000	1.000
	Mean	1.000	1.000	1.000	1.000
288 SNPs	OCL	1.000	1.000	1.000	1.000
	OMN	1.000	1.000	1.000	1.000
	OPT	1.000	1.000	1.000	1.000
	OS	1.000	1.000	1.000	1.000
	OSI	1.000	1.000	1.000	1.000
	Mean	1.000	1.000	1.000	1.000
2145 SNPs	OCL	1.000	0.954	0.939	1.000
	OMN	1.000	1.000	1.000	1.000
	OPT	0.786	1.000	1.000	0.970
	OS	1.000	1.000	1.000	1.000
	OSI	1.000	1.000	1.000	1.000
	Mean	0.957	0.991	0.988	0.994

^1^ PPV = Positive Predictive Value (Precision). ^2^ NPV = Negative Predictive Value.

## Data Availability

The data presented in this study are available on request from the corresponding author due to institutional restrictions.

## Supplementary Materials

The following supporting information can be downloaded at https://www.mdpi.com/article/10.3390/genes17090991/s1, Table S1: Description of the SNPs included in the 288-, 192-, and 96-SNP reduced panels developed for breed assignment in Brazilian locally adapted sheep breeds; Figure S1: Cross-validation (CV) error from ADMIXTURE analyses (K = 2–14) for the complete post-QC BeadChip and the three reduced SNP panels. The minimum CV error was observed at K = 5 for all marker sets; Table S2: Optimized Random Forest hyperparameters for each SNP panel evaluated for breed assignment in Brazilian locally adapted sheep.

## Abbreviations

The following abbreviations are used in this manuscript:

AIMs	Ancestry-informative markers
CI	Confidence interval
CV	Cross validation
EDTA	Ethylenediaminetetraacetic acid
FST	Pairwise Wright’s fixation index
LD	Linkage disequilibrium
NPV	Negative predictive value
OOB	Out-of-bag
PCA	Principal Component Analysis
PPV	Positive predictive value
QC	Quality control
SNP	Single-nucleotide polymorphism
